# Associations of coffee genetic risk scores with consumption of coffee, tea and other beverages in the UK Biobank

**DOI:** 10.1111/add.13975

**Published:** 2017-09-29

**Authors:** Amy E. Taylor, George Davey Smith, Marcus R. Munafò

**Affiliations:** ^1^ MRC Integrative Epidemiology Unit (IEU) University of Bristol Bristol UK; ^2^ UK Centre for Tobacco and Alcohol Studies, School of Experimental Psychology University of Bristol Bristol UK; ^3^ School of Social and Community Medicine University of Bristol Bristol UK

**Keywords:** Caffeine, coffee, drinks, genetics, Mendelian randomization, tea

## Abstract

**Aims:**

To evaluate the utility of coffee‐related genetic variants as proxies for coffee consumption in Mendelian randomization studies, by examining their association with non‐alcoholic beverage consumption (including subtypes of coffee and tea) and a range of socio‐demographic and life‐style factors.

**Design:**

Observational study of the association of genetic risk scores for coffee consumption with different types of non‐alcoholic beverage consumption.

**Setting:**

UK general population.

**Participants:**

Individuals of European ancestry aged 40–73 years from the UK Biobank between 2006 and 2010 (*n* = 114 316).

**Measurements:**

Genetic risk scores were constructed using two, four and eight independent single nucleotide polymorphisms (SNPs) identified in genome‐wide association studies (GWAS) of coffee consumption. Drinks were self‐reported in a baseline questionnaire (all participants) and in detailed 24 dietary recall questionnaires in a subset (*n* = 48 692).

**Findings:**

Genetic risk scores explained up to 0.38, 0.19 and 0.76% of the variance in coffee, tea and combined coffee and tea consumption, respectively. Genetic risk scores demonstrated positive associations with both caffeinated and decaffeinated coffee and tea consumption, and with most subtypes of coffee consumption, but only with standard tea consumption. There was no clear evidence for positive associations with most other non‐alcoholic beverages, but higher genetic risk for coffee consumption was associated with lower daily water consumption. The genetic risk scores were associated with increased alcohol consumption, but not consistently with other socio‐demographic and life‐style factors.

**Conclusions:**

Coffee‐related genetic risk scores could be used as instruments for combined coffee and tea consumption in Mendelian randomization studies. However, associations observed with alcohol consumption require further investigation to determine whether these are due to causal effects of coffee and tea consumption or biological pleiotropy.

## Introduction

Genome‐wide association studies (GWAS) have identified eight independent loci that are associated with coffee consumption at the genome‐wide significance level 1, 2, 3. The two most strongly associated loci identified to date are in or near genes which are involved in caffeine metabolism, namely the cytochrome P450 1A1 and 1A2 (*CYP1A1*/*2*) gene region and aryl‐hydrocarbon receptor gene (*AHR*) 4. CYP1A2 is the enzyme responsible primarily for metabolizing caffeine and AHR affects CYP1A2 activity 4. It is likely that these variants affect coffee consumption through altering rate of caffeine metabolism; there is evidence that the coffee consumption‐increasing alleles of these single nucleotide polymorphisms (SNPs) decrease blood caffeine levels 5, 6. Of note, five of the six additional loci (or close proxies, in or near the following genes: *GCKR*, *ABCG2*, *MLXIPL*, *BDNF*, *EFCAB5*) have also been identified in GWAS of other phenotypes, such as body mass index and smoking initiation 3, 7, 8. Combining variants together in an allele score increases the amount of variance in coffee consumption explained 9, 10. However, there is also evidence that coffee‐related SNPs and genetic risk scores associate more broadly with caffeinated beverage consumption 10 and with decaffeinated coffee intake 3. Better characterization of the coffee and caffeine phenotypes that this genetic risk score capture is important for interpreting the results of analyses that use these as proxies or markers of coffee exposure.

**Figure 1:**
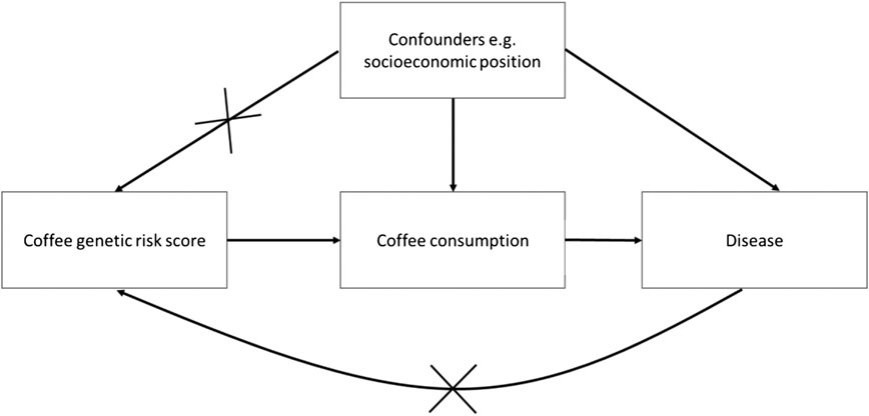
Diagram of Mendelian randomization approach. In Mendelian randomization, genetic variants for coffee consumption are used as instruments for measured coffee consumption to assess if relationships between coffee consumption and disease are likely to be causal. Unlike measured coffee consumption, under the assumptions of Mendelian randomization, genetic variants that influence coffee consumption should not be associated with potential confounding factors and will not be affected by the disease outcome (no reverse causality)

One important method for which coffee genetic risk score may prove useful is Mendelian randomization, which uses genetic variants as proxies for measured exposures to strengthen causal inference 11. Unlike measured coffee consumption genetic variants should, in theory, be independent of confounders that may distort relationships between coffee consumption and should not be affected by reverse causality (see Fig. 1). To be suitable instruments for Mendelian randomization analyses, genetic risk scores for coffee or caffeine consumption should (1) be associated robustly with the exposure of interest, (2) not be associated with potential confounding factors of the exposure–outcome relationship and (3) be associated with the outcome only through the exposure of interest (the exclusion restriction assumption) 12. Biological pleiotropy (i.e. a genetic variant influencing more than one phenotypic trait) can often violate the third assumption in Mendelian randomization studies (although methods for accounting for potential bias due to pleiotropy 13, such as MR Egger 14 have been developed). This can be particularly problematic when exposures are complex behavioural traits such as substance use, given the numerous biological pathways involved and the likelihood that genetic variation in these pathways will have multiple downstream effects.

Given that caffeine is the most widely used psychoactive substance in the world 15, and that consumption of coffee and tea is associated with numerous health outcomes, including cardiovascular disease, diabetes, depression and mortality 16, 17, 18, 19, the use of these genetic risk scores is likely to become more widespread. There is considerable interest in the potential effects of coffee, tea and caffeine consumption on health; to date, coffee‐related variants have been used in Mendelian randomization analyses as instruments for coffee consumption to investigate associations with diabetes 20, cardiovascular risk factors, depression, childhood cognition and Alzheimer's disease 21 and prostate cancer 22. More detailed assessment of how genetic risk scores relate to the exposure of interest (i.e. coffee) and how specific they are as instruments for this exposure is necessary for interpreting the results of these studies correctly and understanding the potential limitations of Mendelian randomization for investigating the causal effects of these traits.

We conducted the analysis in 114 316 individuals from the UK Biobank study, including a subset of 48 692 individuals with 2‐hour dietary recall data, with the aims of determining the associations of coffee‐related genetic risk scores with: (1) non‐alcoholic beverage consumption (including subtypes of coffee and tea) and (2) socio‐demographic and life‐style factors. These analyses extend previously published work conducted in UK Biobank, which presented directions of effect and *P*‐values of these SNPs with coffee and tea intake, but did not investigate associations with subtypes of coffee and tea or other beverages 6.

## Methods

### Design

We calculated polygenic risk scores from a previously published genome‐wide analysis of coffee consumption and tested associations of these with self‐reported dietary, life‐style and socio‐demographic data in the UK Biobank.

### Study population

The UK Biobank (www.ukbiobank.ac.uk) recruited more than 500 000 men and women (aged 40–73 years) between 2006 and 2010 23. Participants attended one of the 22 assessment centres in England, Wales and Scotland, where they provided information on demographic, life‐style factors and medical history through interviews and questionnaires and had physical measurements and blood, urine and saliva samples taken. The full protocol for the study is available online: www.ukbiobank.ac.uk/wp‐content/uploads/2011/11/UK‐Biobank‐Protocol.pdf. The UK Biobank study was approved by the North West Multi‐Centre Research Ethics Committee and all participants provided written informed consent to participate in the UK Biobank study.

### Genetic risk scores

We created genetic risk scores for caffeine consumption using eight SNPs from eight independent loci that reached genome‐wide significance, with coffee consumption in the trans‐ethnic meta‐analysis in the Coffee and Caffeine Genetics Consortium (CCGC) (rs4410790, rs2470893, rs1260326, rs1481012, rs7800944, rs9902453, rs17685, rs6265) 3. We also created a genetic risk score using only variants in or near the strongest two loci, *AHR* (rs4410790) and *CYP1A1/2* (rs2470893), which have been identified in multiple GWAS studies of coffee consumption 1, 2 and a score with four SNPs from loci that reached genome‐wide significance in the European discovery sample and were replicated in the European replication sample (rs4410790, rs2470893, rs7800944, rs17685). Full details of these SNPs are provided in Supporting information, Table S1. Unweighted risk scores were created by adding together the number of coffee consumption‐increasing alleles. Weighted risk scores were created by multiplying each coffee consumption‐increasing allele by the magnitude of its association with coffee consumption in individuals of European ancestry in the discovery sample of the CCGC genome‐wide association study 3 (see Supporting information, Table S1). The UK Biobank sample was not part of the CCGC GWAS sample.

### Tea, coffee and other beverage consumption

Information about usual intake of tea and coffee were assessed as part of the baseline questionnaire, which was administered to all participants during their visit to the initial assessment centre. All UK Biobank participants were asked how many glasses of water they drank each day. For each type of drink, answers were provided on a continuous scale and we excluded individuals reporting drinking > 25 cups/glasses a day. Information on tea, coffee and other non‐alcoholic beverage consumption was also obtained for a subset of participants on up to five occasions from a validated 24‐hour diet recall 24. Full details of the questions asked, the coding of drink consumption and the coding of caffeinated and decaffeinated coffee and tea consumption are provided in the Supporting information.

### Socio‐demographic and life‐style factors

Information on education, socio‐economic deprivation, alcohol consumption and income was collected at baseline. See Supporting information for details.

### Statistical analysis

Analyses were conducted in Stata version 14.2. Associations between genetic risk scores and consumption of each type of beverage were assessed using linear regression, adjusting for age (as a continuous variable), sex and 15 genetic principal components. Robust standard errors were calculated to account for non‐normality of residuals. Weighted risk scores were converted to *Z*‐scores, so associations are per standard deviation (SD) increase. Associations for unweighted risk scores are per additional coffee consumption‐increasing allele. Primary analyses in the full UK Biobank sample were conducted in all individuals (consumers and non‐consumers). Analyses in the subset with dietary data were restricted to consumers of each beverage, as many of the beverages were consumed by only a small proportion of the sample. We also tested for associations with any versus no tea and coffee consumption in the full sample using logistic regression. Where evidence for associations with beverages other than coffee or tea was found, we investigated whether associations might be due to causal effects of coffee or tea consumption by performing inverse variance‐weighted Mendelian randomization and methods which are more robust to the effects of pleiotropy: median‐weighted regression 25 and MR Egger 14. A more detailed explanation of these analyses is provided in the Supporting information.

### Sensitivity analyses

Smoking is known to increase caffeine metabolism through induction of CYP1A2 26, so we performed a sensitivity analysis in the full sample stratified by smoking status (never, former and current). As the initial GWAS release of the UK Biobank contains data from a nested case–control study, participants were selected on the basis of lung function and smoking status (UK BiLEVE) 27; we also repeated analyses excluding these individuals.

## Results

### Description of study population

A total of 114 316 unrelated individuals of European ancestry were included in the analysis of coffee and tea captured at the initial UK Biobank assessment centre (see Supporting information, Fig. S1). Dietary recall data were available for a subset of 48 692 of these individuals (Table 1). Prevalence of tea and coffee consumption was high (85% of individuals were tea consumers and 78% coffee consumers in the full sample). Coffee and tea intake were correlated negatively (*r* = −0.33 in all individuals and *r* = −0.17 in individuals consuming both tea and coffee; *P*‐values < 0.001).

**Table 1:** Description of study samples.

	Full sample (n = 114 316)		Subset with dietary recall data (n = 48 692)	
Male: *n* (%)	54 024	47.26	22 789	46.80
Age: mean (SD)	56.9	7.92	56.49	7.79
Education				
None	20 615	18.20	4559	9.40
NVQ/CSE/A‐levels	40 898	36.10	16 578	34.17
Degree/professional	51 770	45.70	27 377	56.43
Any tea consumption	96 765	84.65	41 324	84.98
Tea (cups per day)	3	1,5	3	1,5
Any coffee consumption	90 005	78.73	39 298	80.77
Coffee (cups per day)	2	0.5,3	2	0.5,3
Smoking				
Never	61 179	53.65	27 389	56.35
Former	39 006	34.21	16 848	34.66
Current	13 844	12.14	4371	8.99
Questionnaires completed: *n* (%)				
1			19 093	39.21
2			11 160	22.92
3			9895	20.32
4			7198	14.78
5			1346	2.76

SD = standard deviation; NVQ = National Vocational Qualification; CSE = Certificate of Secondary Education; A‐level = Advanced level.

### Variance explained in coffee and tea consumption by genetic risk score

In the first release of the UK Biobank sample with genetic data, there was evidence that each of the SNPs apart from rs6265 were associated with coffee consumption (either in the full sample or only in coffee consumers) in the same direction as described previously (Supporting information, Fig. S2) 3 .The two SNP, four SNP and eight SNP genetic risk scores were associated with coffee and tea intake (see Table 2). The risk scores explained up to 0.38% of the variance in coffee consumed per day, up to 0.19% of the variance in tea consumed per day and up to 0.76% of the variance in tea and coffee combined. The weighted eight SNP score explained the highest proportion of the variance for both tea and coffee (Table 2). However, the majority of this variance was explained by the two strongest SNPs (rs4410790 and rs2470893). Variance explained by the additional six SNPs alone is shown in Supporting information, Table S2. The eight SNP genetic risk score explained a very similar amount of variance to the four SNP genetic risk score.

**Table 2:** Associations of eight and two single nucleotide polymorphism (SNP) genetic risk scores with tea and coffee consumption (cups per day).

	n	Unweighted b beta (95% CI)	R‐squared a	Weighted c beta (95% CI)	R‐squared a
Cups of coffee per day (including decaffeinated)					
2 SNP score	114 316	0.12 (0.11, 0.13)	0.30% (0.24, 0.35%)	0.11 (0.10, 0.13)	0.30% (0.24, 0.35%)
4 SNP score	113 425	0.09 (0.08, 0.10)	0.33% (0.27, 0.39%)	0.13 (0.11, 0.14)	0.36% (0.30,0.42%)
8 SNP score	111 469	0.06 (0.06, 0.07)	0.28% (0.23,0.35%)	0.13 (0.12, 0.14)	0.38% (0.32, 0.46%)
Cups of tea per day					
2 SNP score	114 316	0.12 (0.10, 0.13)	0.16% (0.12, 0.20%)	0.11 (0.10, 0.13)	0.16% (0.12, 0.20%)
4 SNP score	113 425	0.09 (0.07, 0.10)	0.16% (0.12, 0.20%)	0.12 (0.11, 0.14)	0.19% (0.15, 0.24%)
8 SNP score	111 469	0.06 (0.05, 0.07)	0.13% (0.09, 0.17%)	0.12 (0.11, 0.14)	0.19% (0.14, 0.24%)
Cups of tea and coffee per day					
2 SNP score	114 316	0.24 (0.22, 0.26)	0.62% (0.53, 0.69%)	0.23 (0.21,0.24)	0.61% (0.53, 0.69%)
4 SNP score	113 425	0.18 (0.17, 0.19)	0.65% (0.56, 0.72%)	0.25 (0.23, 0.27)	0.74% (0.66, 0.82%)
8 SNP score	111 469	0.12 (0.11, 0.13)	0.54% (0.46, 0.62%)	0.25 (0.24,0.27)	0.76% (0.67, 0.86%)

Analyses include consumers and non‐consumers. Eight SNP scores had missing genotype information for 2847 individuals.

a Calculated from residuals of risk score on genetic principal components, regressed on coffee/tea/tea and coffee per day.

b Associations in cups per day per coffee consumption‐increasing allele, adjusted for age, sex and genetic principal components.

c Associations in cups per day per standard deviation (SD) increase in genetic risk score, adjusted for age, sex and genetic principal components. CI = confidence interval.

There was evidence that the caffeine genetic risk scores were associated with higher odds of drinking any coffee compared to no coffee [odds ratio (OR) per SD increase in eight SNP‐weighted genetic risk score = 1.06, 95% confidence interval (CI) = 1.05, 1.08]. However, there was no clear evidence that the genetic risk scores were associated with consuming either any tea or any tea or coffee combined (Supporting information, Table S3). Associations between the genetic risk scores and coffee and tea combined were similar at low and medium levels of coffee/tea consumption. In high consumers (> 10 cups per day) magnitudes of effect were lower, but estimates were imprecise (Supporting information, Table S4).

### Associations of genetic risk score with coffee, tea and other beverage consumption

In analyses restricted to consumers of each type of drink, in the full sample each additional coffee consumption‐increasing allele of the two SNP genetic risk score was associated with a 0.12 increase in the number of cups of coffee consumed per day (95% CI = 0.10, 0.13), a 0.14 increase in the number of cups of tea consumed per day (95% CI = 0.13, 0.16) and a 0.24 increase in the number of cups of tea and coffee combined (95% CI = 0.22, 0.26). We observed a negative association with water consumption; each additional coffee consumption‐increasing allele was associated with consuming 0.06 fewer glasses of water per day (95% CI = –0.07, −0.04).

**Figure 2:**
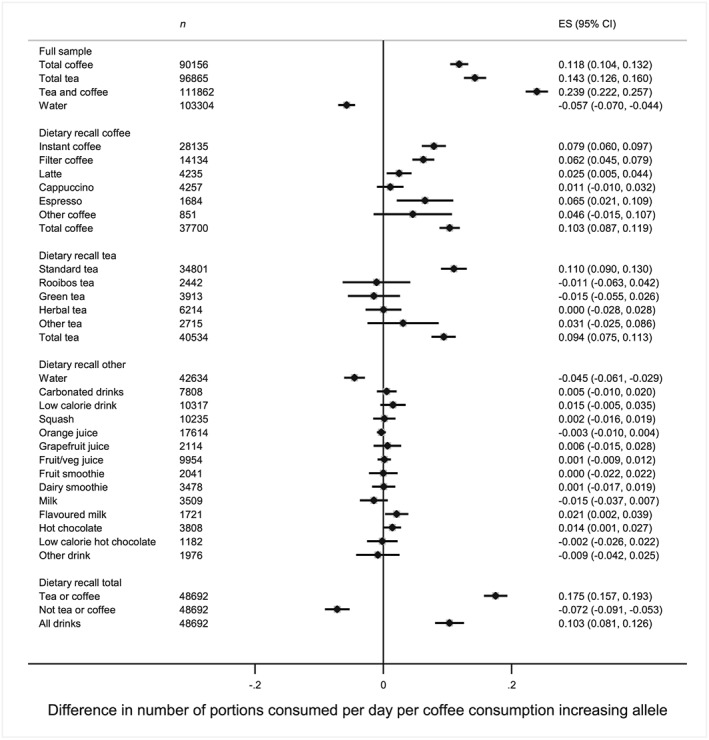
Associations between the two single nucleotide polymorphism (SNP) genetic risk scores and types of drink in the full sample and dietary recall subset. Analyses restricted to consumers of each drink, apart from the dietary recall total which included all respondents. Adjusted for age, sex and principal genetic components

Similar patterns were observed in the subset of individuals with dietary recall data; the two SNP‐unweighted genetic risk score was associated positively with most types of coffee consumption (Fig. 2). Each additional caffeine consumption‐increasing allele was associated with higher consumption of instant, filter, latte, decaffeinated and total coffee. For cappuccino and other coffee, point estimates were in the positive direction but there was little statistical evidence for associations. The genetic risk score was also associated with increased standard tea but not with rooibos, green tea or herbal tea. As observed in the full sample, the genetic risk score was associated negatively with water consumption. The genetic risk score was associated positively with combined coffee and tea consumption (0.18 additional portions consumed per additional coffee consumption‐increasing allele, 95% CI = 0.16, 0.19), but negatively with non‐coffee and tea consumption (0.07 fewer portions consumed per additional coffee consumption‐increasing allele, 95% CI = –0.09, −0.05). Results using the four and eight SNP‐weighted genetic risk score were similar (Supporting information, Figs S3 and S4). There was no clear evidence that the genetic risk score was associated with specific consumption of any of the other non‐tea or coffee beverages, apart from suggestive evidence for increased consumption of flavoured milk and hot chocolate. However, evidence for these associations was weaker with the four and eight SNP score.

**Figure 3:**
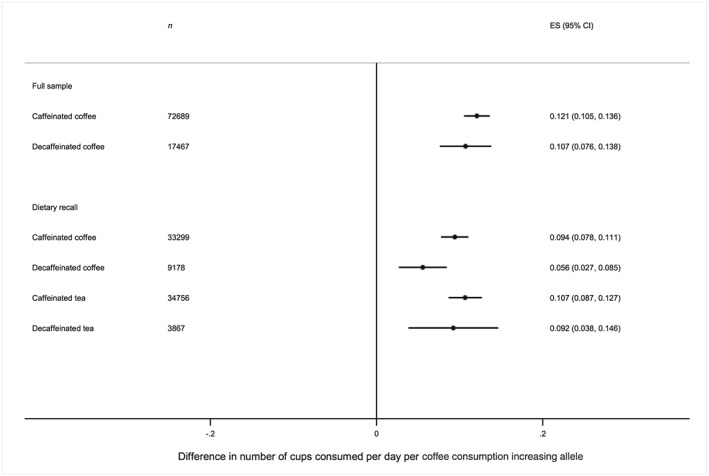
Associations between the two single nucleotide polymorphism (SNP) genetic risk scores and caffeinated and decaffeinated coffee. Analyses restricted to consumers of each drink. Adjusted for age, sex and principal genetic components

The two SNP genetic risk score was positively associated with both caffeinated and decaffeinated tea and coffee consumption (Fig. 3). Point estimates for decaffeinated coffee consumption and caffeinated coffee consumption were similar in the full UK Biobank sample, but were smaller for decaffeinated coffee consumption (beta 0.06, 95% CI = 0.03, 0.09) than for caffeinated consumption (beta 0.09, 95% CI = 0.08, 0.11) in the dietary recall sample. For tea consumption, point estimates were similar for both caffeinated and decaffeinated in the dietary recall study. Similar patterns were observed for the eight and four SNP scores (see Supporting information, Figs S5 and S6).

### Association of genetic risk score with demographic and life‐style factors

To test the second assumption of Mendelian randomization analysis, we investigated associations between the instrument and potential confounding factors. We found no clear evidence that the genetic risk scores were associated with age, education levels, income or level of deprivation (Table 3). However, there was evidence for a positive association with both frequency of alcohol consumption (OR for daily versus non‐daily consumption per SD increase in eight SNP genetic risk score 1.02, 95% CI = 1.01, 1.04) and weekly alcohol consumption among alcohol consumers. The two SNP genetic risk score also showed suggestive evidence of being associated with current smoking (OR for current smoking per coffee consumption‐increasing allele 0.98 (95% CI = 0.96, 1.00) and sex (OR for female sex per coffee consumption‐increasing allele 0.99 (95% CI = 0.97, 1.00), although evidence for these associations was weaker with the eight SNP genetic risk score. Associations between genetic risk score and demographic and life‐style factors showed some differences in samples stratified into high, medium, low and non‐coffee and tea consumers (Supporting information, Figs S6 and S7).

**Table 3:** Association between genetic risk scores and demographic factors.

	2 SNP genetic risk score^a^			4 SNP genetic risk score^b^			8 SNP genetic risk score^b^		
	n	Beta/OR (95% CI)	P‐value	n	Beta/OR (95% CI)	P‐value	n	Beta/OR (95% CI)	P‐value
Female sex	114 316	0.986 (0.974, 0.998)	0.03	113 425	0.987 (0.976, 0.999)	0.03	111 469	0.990 (0.979, 1.002)	0.10
Age (years)	114 316	0.03 (−0.02, 0.08)	0.22	113 425	0.02 (−0.02, 0.07)	0.35	111 469	0.03 (−0.02, 0.07)	0.27
Townsend deprivation index	114 167	−0.005 (−0.023, 0.013)	0.58	113 278	−0.009 (−0.026, 0.008)	0.30	111 323	−0.004 (−0.021, 0.013)	0.65
Degree/ professional qualification (yes/no)	113 283	0.995 (0.983, 1.007)	0.40	112 401	0.996 (0.984, 1.008)	0.48	110 463	0.992 (0.981, 1.004)	0.20
Household income	98 890	−0.006 (−0.014, 0.002)	0.13	98 108	−0.002 (−0.009, 0.005)	0.61	96 414	−0.006 (−0.013, 0.001)	0.12
Current smoking (yes/no)	114 029	0.981 (0.962, 0.999)	0.04	113 138	0.983 (0.966, 1.001)	0.07	111 186	0.988 (0.970, 1.006)	0.17
Daily alcohol consumption (yes/no)	114 254	1.016 (1.001, 1.032)	0.03	113 363	1.017 (1.003, 1.032)	0.02	111 407	1.024 (1.010, 1.039)	0.001
Weekly alcohol consumption (units)^c^	87 603	1.009 (1.001, 1.016)	0.02	86 933	1.010 (1.003, 1.017)	0.006	85 410	1.015 (1.008, 1.022)	<0.001

Associations with sex, education, smoking and alcohol consumption are odds ratios from logistic regression. Associations with age, deprivation, income are beta coefficients from linear regression. Income is measured in the following categories: less than £18 000, £18 000–30 999, £31 000–£51 999, £52 000–£100 000, greater than £100 000. Analyses adjusted for 15 genetic principal components.

a Associations per coffee consumption‐increasing allele.

b Associations are per standard deviation (SD) increase in genetic risk score.

c Only among individuals reporting some alcohol consumption. Values were log‐transformed and coefficients exponentiated to represent ratios of geometric means. SNP = single nucleotide polymorphism; OR = odds ratio; CI = confidence interval. All analyses adjusted for genetic principal components.

### Sensitivity analyses

There was no strong evidence that associations between coffee genetic risk score and coffee and tea consumption differed in never, former or current smokers (Supporting information, Fig. S8). Restriction of analyses to individuals not in the UK BiLEVE sample made little difference to the observed associations (data not shown).

Results from inverse variance‐weighted Mendelian randomization analysis, MR Egger and median‐weighted regression assessing the association between the eight SNP genetic risk score and water consumption were highly consistent, providing some support for a negative causal effect of coffee/tea consumption on total non‐coffee and tea consumption and water consumption (see Supporting information, Table S5). In contrast, while results from median‐weighted regression supported a potential causal effect of coffee consumption on alcohol intake, results from MR Egger analyses suggested potential horizontal or biological pleiotropy (Supporting information, Table S6).

## Discussion

We have confirmed associations of genetic risk score for coffee consumption using variants identified in published GWAS 1, 2, 3, with amount of consumption of both coffee and tea in a large sample of older adults of European ancestry from the United Kingdom. Using a more detailed measure of beverage consumption, the 24‐hour dietary recall, we found that the genetic risk score was associated with most subtypes of coffee consumption, and with black tea but not herbal tea consumption. These scores were also associated with decreased consumption of water and total drinks excluding tea and coffee.

Associations of the genetic risk scores with both coffee and tea support the use of coffee genetic risk scores as instruments for amount of coffee and tea consumed (and probably caffeine consumption in general) rather than as specific markers of coffee consumption. The variance explained in combined coffee and tea consumption was 0.54–0.76% compared to 0.13–0.38% for coffee or tea alone. This has been shown previously in a study of women from the United Kingdom 10 and is unsurprising, given that the two strongest coffee‐related variants in the risk score (*AHR* and *CYP1A1/2*) are in or near genes in caffeine metabolizing pathways. In addition, the genetic risk scores influenced whether individuals were coffee consumers but not whether they were tea consumers. Lack of association with any tea consumption may simply reflect the widespread consumption of tea in the United Kingdom (85% in this study), but could also be explained by the lower caffeine content of tea compared to coffee.

The effect sizes we observed with coffee consumption were similar to those observed in previous GWAS of coffee consumption 2, 3. Our data suggest that using the four SNP‐weighted genetic risk score increases power to detect associations with caffeinated drink consumption over the two SNP genetic risk score (weighted or unweighted), but only by a relatively small amount. Using SNPs from all eight loci explained a similar amount of variance to the four SNP score, suggesting that there may be little added benefit to using the four additional SNPs to improve the power of analyses in European populations. The fact that the association of the *BDNF* SNP rs6265 with coffee consumption did not replicate in UK Biobank (and also did not replicate in the European sample of the GWAS [3]) suggests that this locus does not associate robustly with coffee consumption in European samples. The two SNP risk score may be a more appropriate instrument for coffee or caffeine consumption for some Mendelian randomization analyses, given that the other genetic variants are known to be associated with other non‐caffeine‐related phenotypes. However, it is also important to note that CYP1A2 metabolizes xenobiotic substrates other than caffeine 28, so SNPs in or near *CYP1A1/2* and *AHR* may have downstream effects which do not act through caffeine.

Our analysis of the 24‐hour dietary recall data allowed us to explore the specificity of these associations to subtypes of tea and coffee as well as other types of beverage consumption. In general, we found consistent evidence for positive associations between the coffee‐related genetic risk scores and subtypes of coffee (e.g. filter, latte, espresso), but associations with tea were limited to standard tea and did not include other subtypes (rooibos, herbal, green). There was limited evidence for associations with other types of beverage (with the exception of water). Given the variation in consumption of different types of coffee and tea between countries 29 and by demographic factors (e.g. age) 4, it is possible that these associations will be context‐specific. This therefore highlights an important potential source of heterogeneity that may arise between studies when using these risk scores.

The genetic risk scores associated with both caffeinated and decaffeinated coffee and tea consumption in UK Biobank. Associations of *AHR* variants with decaffeinated coffee consumption were also reported in the CCGC GWAS 3. This is likely to be explained by a continuation of coffee and tea drinking behaviour in individuals who have switched from caffeinated to decaffeinated coffee and tea consumption. However, we did not observe associations with herbal, green or rooibos tea in UK Biobank; it is possible that these drinks are not consumed in high enough quantities or that they are not direct replacements for caffeinated beverages. Importantly, it is likely that there is some misclassification of decaffeinated tea and coffee consumption due to the way in which individuals were asked about decaffeinated drink consumption in UK Biobank (only being able to say they drank all decaffeinated, all caffeinated or a mixture). Therefore, some caution must be exercised when interpreting these results.

To our knowledge, the negative association between coffee‐related genetic variants and water consumption has not been reported previously. The negative association was also observed for overall non‐tea and non‐coffee drink consumption. We think that the most parsimonious explanation for this finding is that this is a downstream effect of increased coffee and tea consumption; individuals drinking more coffee and tea consume fewer other drinks. This is consistent with fluid homeostasis, whereby the body maintains fluid balance within a healthy range. Our findings from Mendelian randomization analysis also provide some support for this being a downstream effect of coffee/tea consumption (i.e. vertical or mediated pleiotropy); MR Egger and median‐weighted regression analysis both provided some evidence for causal negative effects of coffee consumption on water and total other beverage consumption.

We found some evidence within UK Biobank for associations with other traits, most notably alcohol consumption. This could be of concern for use of these risk scores as proxies for coffee or caffeine consumption in Mendelian randomization studies, as this would potentially violate the assumption of no horizontal or biological pleiotropy. A previous Mendelian randomization study using variants in *CYP1A1/2* and *AHR* did not find clear evidence for associations with potential confounders 20, but did not investigate alcohol consumption. However, one of the coffee‐related variants, rs1260326 (in *GCKR*), has been identified as a novel locus for alcohol consumption in a GWAS conducted in the initial UK Biobank release 30. Further work is required to determine whether this represents a true association and, if so, whether this effect operates through coffee/tea consumption or through an independent pathway—the latter would violate the assumptions of Mendelian randomization. Our finding that there was some difference in associations between risk scores and demographic and life‐style factors in groups with different levels of consumption suggests that stratifying on level of consumption when using these scores could cause collider stratification bias (where selection into a sample can induce confounding) 31, 32.

There are several limitations to this analysis that should be considered. First, these analyses have been conducted in individuals of European ancestry and may not be generalizable to other ethnicities. As discussed above, variation in type of caffeine consumption by population may also limit the generalizability of these results. Secondly, the UK Biobank had a low response rate (approximately 5%), and is not likely to be representative of individuals of this age group in the United Kingdom 33. Thirdly, there is likely to be measurement error in the reporting of beverage consumption. In the main questionnaire, individuals could report consumption of tea and coffee as the exact number of cups consumed per day, but in the dietary recall study individuals could only report consumption up to a maximum of six or more portions per day. Although patterns of association were very similar for tea and coffee between the two, this restriction of the maximum value is likely to impact upon the magnitude of associations, particularly when summing across categories. Therefore, effect sizes should be interpreted with caution. Fourthly, we were unable to specifically distinguish other forms of caffeinated beverage consumption in UK Biobank. It is likely that the carbonated and low‐calorie drink categories contain some caffeine‐containing beverages (e.g. cola). Finally, this study does not assess the association of these risk scores with blood caffeine levels. As discussed above, variants in or near *CYP1A1/2* and *AHR* which increase coffee consumption decrease plasma caffeine 5, 6. A recent analysis including data from UK Biobank has also demonstrated that the SNPs in *GCKR*, *EFCAB5* and *POR* also associate in opposing directions with coffee consumption and plasma caffeine metabolites 6.

Genetic risk scores for coffee consumption are already being used as a tool to explore the causal effects of coffee consumption 20, 22 and their use is likely to become more widespread, given the range of health conditions with which coffee consumption is associated. We have demonstrated that genetic risk scores compiled using variants from coffee consumption GWAS associate more broadly with caffeine‐containing beverages (or decaffeinated versions of these beverages). What these variants relate to within each specific analysis sample will be important for interpreting the results of Mendelian randomization analyses. Finally, association of these genetic risk scores with non‐caffeine‐related phenotypes, e.g. alcohol consumption, may invalidate their use to assess the downstream health effects of caffeine consumption.

## Declaration of interests

A.E.T is in receipt of a GRAND award from Pfizer, which is unrelated to the submitted work.

## Supporting information


**Table S1** Single nucleotide polymorphisms (SNPs) used in the caffeine genetic risk scores.
**Table S2** Association of six single nucleotide polymorphism (SNP) scores with coffee and tea.
**Table S3** Associations between coffee variants and any versus no tea or coffee consumption.
**Table S4** Associations between coffee variants and combined coffee and tea consumption stratified by number of cups of coffee and tea consumed per day.
**Table S5** Mendelian randomization analysis of the association of coffee genetic risk scores with water and total non‐coffee or tea beverage consumption.
**Table S6** Mendelian randomization analysis of the association of coffee genetic risk scores with daily alcohol consumption.
**Figure S1** Flow‐chart of study population.
**Figure S2** Associations of individual single nucleotide polymorphisms with coffee consumption.
**Figure S3** Association between eight single nucleotide polymorphism (SNP)‐weighted genetic risk score and types of drink in the full sample and dietary recall subset.
**Figure S4** Association between four single nucleotide polymorphism (SNP)‐weighted genetic risk score and types of drink in the full sample and dietary recall subset.
**Figure S5** Associations between the eight single nucleotide polymorphism (SNP) genetic risk score and caffeinated and decaffeinated coffee.
**Figure S6** Associations between the four single nucleotide polymorphism (SNP) genetic risk score and caffeinated and decaffeinated coffee.
**Figure S7** Association between genetic risk scores and continuous demographic and life‐style factors stratified by combined coffee and tea consumption.
**Figure S8** Association between genetic risk scores and binary demographic and life‐style factors stratified by combined coffee and tea consumption.
**Figure S9** Association between genetic risk scores and tea and coffee consumption stratified by smoking status.
