# Supplementary material for: Associations of coffee genetic risk scores with consumption of coffee, tea and other beverages in the UK Biobank
Source: Addiction. 2017 Sep 29;113(1):148–57. doi: 10.1111/add.13975 (PMC5765424; doi:10.1111/add.13975)
Supplement: Supplementary file 1 — Table S1 Single nucleotide polymorphisms (SNPs) used in the caffeine genetic risk scores. Table S2 Association of six single nucleotide polymorphism (SNP) scores with coffee and tea. Table S3 Associations between coffee variants and any versus no tea or coffee consumption. Table S4 Associations between coffee variants and combined coffee and tea consumption stratified by number of cups of coffee and tea consumed per day. Table S5 Mendelian randomization analysis of the association of coffee genetic risk scores with water and total non‐coffee or tea beverage consumption. Table S6 Mendelian randomization analysis of the association of coffee genetic risk scores with daily alcohol consumption. Figure S1 Flow‐chart of study population. Figure S2 Associations of individual single nucleotide polymorphisms with coffee consumption. Figure S3 Association between eight single nucleotide polymorphism (SNP)‐weighted genetic risk score and types of drink in the full sample and dietary recall subset. Figure S4 Association between four single nucleotide polymorphism (SNP)‐weighted genetic risk score and types of drink in the full sample and dietary recall subset. Figure S5 Associations between the eight single nucleotide polymorphism (SNP) genetic risk score and caffeinated and decaffeinated coffee. Figure S6 Associations between the four single nucleotide polymorphism (SNP) genetic risk score and caffeinated and decaffeinated coffee. Figure S7 Association between genetic risk scores and continuous demographic and life‐style factors stratified by combined coffee and tea consumption. Figure S8 Association between genetic risk scores and binary demographic and life‐style factors stratified by combined coffee and tea consumption. Figure S9 Association between genetic risk scores and tea and coffee consumption stratified by smoking status. [file ADD-113-148-s001.docx]

**Supplementary Methods**

**Assessment of tea and coffee in the UK Biobank**

In the baseline UK Biobank questionnaire, participants were asked the following questions about tea and coffee consumption: 1) How many cups of tea (black and green) do you drink each day? (answers provided on a continuous scale), 2) How many cups of coffee (including decaffeinated) do you drink each day? (answers provided on a continuous scale), and 3) Which type of coffee do you usually drink? (decaffeinated coffee/instant coffee/ground coffee/other/do not know/prefer not to answer). Individuals reporting drinking some tea or coffee but less than one cup per day were coded as drinking 0.5 cups per day. For the final question, participants who drank more than one type were asked to select the type that they drank most often.

The 24 hour dietary recall questionnaire was administered at the last station of the assessment centre visit towards the end of recruitment and also emailed to all participants with known email addresses (N = 320,000) on four separate occasions between February 2011 and April 2012. For full details see: <http://biobank.ctsu.ox.ac.uk/crystal/docs/DietWebQ.pdf>. The questionnaire asked about consumption of about 200 commonly consumed items. Participants were asked how many times they consumed specific drinks on the previous day. For each type of drink, they could respond with the following options: None, ½, 1, 2, 3, 4, 5, 6+. Participants were asked if they drank any coffee the previous day, and if so, how many mugs/cups of instant, filter/Americano/cafetiere, cappuccino, latte, espresso or other coffee drinks they had consumed. For each type of coffee, they could respond with the following options: None, ½, 1,2,3,4,5,6+. They were then asked to say if it was decaffeinated coffee to which they could answer no, yes or varied. Participants were asked if they had drank any tea or infusions yesterday and if so, how many mugs/cups of standard tea, rooibos/redbush, green tea, herbal or fruit tea, other tea or infusion they had consumed. For each type of tea, they could respond with the following options: None, ½, 1, 2, 3, 4, 5, 6+. Participants were asked to say if their standard tea was decaffeinated to which they could answer no, yes or varied.

**Other beverage consumption**

In the baseline questionnaire, all UK Biobank participants were asked how many glasses of water they drank each day (answers were provided on a continuous scale). We excluded individuals reporting drinking >25 glasses a day. In the 24 hour diet recall, participants were asked about consumption of the following drinks: water (glass/beaker), low calorie or diet drinks (glass/can), carbonated drinks (glass/can), fruit drinks/J_2_0/squash/cordial (glass/carton/250ml), pure orange juice (glass/carton/250ml), grapefruit juice (glass/carton/250ml), other pure fruit/vegetable juice (glass/carton/250ml), fruit smoothie(glass/carton/250ml), dairy/yoghurt based smoothie (glass/carton/250ml), milk (glass/carton/250ml), yogurt drinks/flavoured milk/milkshakes (glass/carton/250ml), low calorie hot chocolate (mug/cup), hot chocolate (mug/cup), other drinks (glass/mug/cup). For each type of drink, the participants could respond with the following options: None, ½, 1, 2, 3, 4, 5, 6+.

**Calculation of caffeinated and decaffeinated tea and coffee consumption**

In the baseline questionnaire, individuals reporting that the coffee type that they drank most often was decaffeinated were assumed to be decaffeinated coffee drinkers. There was no question about decaffeinated tea consumption in the baseline questionnaire. In the dietary recall data, individuals were asked a single question “Was it decaffeinated coffee?” with reference to consumption of all types of coffee. They were able to respond: No, yes or varied. For individuals responding no all coffee was assumed to be caffeinated. For individuals responding yes, all coffee was assumed to be decaffeinated and for individuals responding varied, we assumed half of their consumption was caffeinated and half decaffeinated. For tea consumption, individuals were asked if their standard tea was decaffeinated and were able to respond: No, yes or varied. For individuals responding no all standard tea was assumed to be caffeinated. For individuals responding yes all standard tea was assumed to be decaffeinated, and for individuals responding varied we assumed half of their standard tea consumption was caffeinated and half decaffeinated.

**Calculating average consumption for the 24 dietary recall**

For the dietary recall data, information on dietary consumption in the previous 24 hours was available for each participant for up to 5 occasions. Therefore we calculated an average consumption for each drink by dividing the total number of times the drink was consumed by the number of times the individual completed the dietary recall questionnaire. We assigned individuals reporting drinking 6 or more drinks per day a value of 7.5, as this was the mean number of cups of tea/coffee drank by individuals consuming more than 5 cups per day in the main questionnaire. Total tea and coffee consumption was calculated by adding up the average number of cups of all types of tea and coffee consumed by each participant. Total consumption of non-tea and coffee were calculated by adding up the average number of portions of each non-tea or coffee beverage consumed.

**Covariates**

Information on age, highest educational qualification (College or University degree, A levels/AS levels (examinations at age 18 years), O levels/GCSEs/CSEs (examinations at age 16 years), NVQ or HND or HNC (vocational qualifications), Other professional qualifications), household income (< £18,000, £18,000 to 30,999, £31,000 to £51,999, £52,000 to £100,000, > £100,000), current smoking and frequency of alcohol consumption were self-reported at the baseline assessment centre. Participants were classified as daily alcohol consumers if they answered that they consumed alcohol “Daily or almost daily” to the question “About how often do you drink alcohol?”. A Townsend deprivation index measure for each individual was calculated based on their postcode from data from the previous national census. This measure is based on unemployment, non-car ownership, non-home ownership, and household overcrowding, with higher values representing greater deprivation.

Individuals reporting at least weekly drinking were asked how many measures of red wine (glasses)/white wine or champagne (glasses)/beer or cider(pints)/spirits(standard measures) /fortified wine (glasses)/other alcoholic drinks (glasses) they consumed in an average week. Individuals reporting less than monthly drinking were asked how many measures of red wine (glasses)/white wine or champagne (glasses)/beer or cider(pints)/spirits(standard measures) /fortified wine (glasses)/other alcoholic drinks (glasses) they consumed in an average month. Each measure was assigned the value of 1 unit of alcohol apart from pints of beer and cider which were assigned 2 units. An average weekly intake was then calculated for each person. For monthly alcohol consumers, values were multiplied by 12 and then divided by 52.

**Genetic data**

DNA was extracted from blood samples using the Promega Maxwell 16 Blood DNA Purification Kit (AS1010), which uses magnetic bead technology to purify the DNA. An initial sample of 152,249 individuals were genotyped for 641,018 SNPs, some using the Affymetrix UK BiLEVE Axiom array (N = 50,005) and some using the Affymetrix UK Biobank Axiom array (N = 102,244). Imputation was conducted using the 1000 genomes Phase 3 and UK10k reference panels. After quality control, just over 73 million SNPs were available for analysis. The analysis sample was restricted to unrelated individuals, based on a threshold of 0.05 estimated from genetic kinships, and to individuals of European genetic ancestry using principal components analyses (PCA). Best guess genotypes from imputation filtered on a minor allele frequency of 0.01 and an info score of 0.8 and were used in these analyses.

**Statistical analyses**

**Inverse variance weighted Mendelian randomisation, median weighted regression and MR Egger**

To estimate the causal association between coffee consumption and total beverage consumption excluding tea and coffee and water consumption, we performed inverse variance weighted Mendelian randomisation analysis. The magnitude of association of each individual SNP with the outcome (total beverage consumption excluding tea and coffee, water) was divided by the magnitude of association of each SNP with the exposure (coffee consumption, obtained from CCGC GWAS) and combined in an inverse variance weighted meta-analysis. MR-Egger regression provides a test of directional (unbalanced) pleiotropy (the intercept) and a valid test of the causal null hypothesis (the slope) (1). Median weighted regression provides a valid estimate of the causal effect when up to 50% of the genetic variants are not valid instruments (2). These analyses were performed using the mregger and mrmedian commands in Stata version 14.1.

**References**

1. Bowden J., Davey Smith G., Burgess S. Mendelian randomization with invalid instruments: effect estimation and bias detection through Egger regression, International journal of epidemiology 2015: 44: 512-525.

2. Bowden J., Davey Smith G., Haycock P. C., Burgess S. Consistent Estimation in Mendelian Randomization with Some Invalid Instruments Using a Weighted Median Estimator, Genetic epidemiology 2016: 40: 304-314.

**Table S1. SNPs used in the caffeine genetic risk scores.**

| **SNP number** | **Gene** | **N** | **Effect allele** | **Effect allele frequency** | **HWE P-value** | **Directly genotyped or imputed** | **Imputation accuracy** | **Beta value for genetic risk score^1^** |
| --- | --- | --- | --- | --- | --- | --- | --- | --- |
| rs4410790 | *AHR* | 114,316 | C | 0.63 | 0.39 | Imputed | 1 | 0.14 |
| rs2470893 | *CYP1A1* | 114,316 | T | 0.33 | 0.75 | Imputed | 1 | 0.12 |
| rs1260326 | *GCKR* | 114,316 | C | 0.61 | 0.25 | Imputed | 1 | 0.04 |
| rs1481012 | *ABCG2* | 112,757 | A | 0.89 | 0.48 | Imputed | 0.9845 | 0.06 |
| rs7800944 | *MLXIPL* | 113,425 | C | 0.29 | 0.56 | Imputed | 0.9954 | 0.05 |
| rs9902453 | *EFCAB5* | 113,813 | G | 0.45 | 0.08 | Imputed | 0.99784 | 0.04 |
| rs17685 | *POR* | 114,316 | A | 0.28 | 0.16 | Imputed | 1 | 0.07 |
| rs6265 | *BDNF* | 114,316 | C | 0.81 | 0.84 | Imputed | 1 | 0.05 |

1. From CCGC GWAS meta-analysis, Molecular Psychiatry (2015), 20, 647-656.

**Table S2. Association of 6 SNP score with coffee and tea**

|  | **N** | **Unweighted^1^**  **Beta (95% CI)** | **R-squared^3^** | **Weighted^2^**  **Beta (95% CI)** | **R-squared^3^** |
| --- | --- | --- | --- | --- | --- |
| **Cups of coffee per day (including decaffeinated)** | | | | | |
| 6 SNP score | 111,469 | 0.04  (0.03, 0.05) | 0.08%  (0.05, 0.11%) | 0.07  (0.05, 0.08) | 0.1%  (0.06, 0.14%) |
| **Cups of tea per day** | | | | | |
| 6 SNP score | 111,469 | 0.03  (0.02, 0.04) | 0.03%  (0.02, 0.04%) | 0.05  (0.04, 0.07) | 0.04%  (0.03, 0.05%) |
| **Cups of tea and coffee per day** | | | | | |
| 6 SNP score | 111,469 | 0.07  (0.06, 0.08) | 0.14%  (0.10, 0.18%) | 0.12  (0.10,0.14) | 0.17%  (0.13, 0.22%) |

Analyses include consumers and non-consumers.

1. Associations per coffee consumption increasing allele, adjusted for age, sex and genetic principal components

2. Associations are per SD increase in genetic risk score, adjusted for age, sex and genetic principal components

3. Calculated from residuals of risk score on genetic principal components, regressed on coffee/tea/tea and coffee per day.

**Table S3. Associations between coffee variants and any vs no tea or coffee consumption.**

|  | **Yes (N)** | **No (N)** | **Odds Ratio (95% CI) Unweighted** | **Odds Ratio (95% CI) Weighted** |
| --- | --- | --- | --- | --- |
| **Any tea consumption** | | | | |
| 2 SNP score | 96,765 | 17,551 | 0.99 (0.98, 1.01) | 0.99 (0.98, 1.01) |
| 8 SNP score | 94,370 | 17,099 | 0.99 (0.99, 1.00) | 0.99 (0.98, 1.01) |
| **Any coffee consumption (including decaf)** | | | | |
| 2 SNP score | 90,005 | 24,311 | 1.07 (1.05, 1.08) | 1.06 (1.05, 1.08) |
| 8 SNP score | 87,773 | 23,696 | 1.03 (1.02, 1.04) | 1.06 (1.05, 1.08) |
| **Any tea or coffee consumption** | | | | |
| 2 SNP score | 111,862 | 2,454 | 1.04 (0.99, 1.08) | 1.03 (0.99, 1.08) |
| 8 SNP score | 109,083 | 2,386 | 1.02 (0.99, 1.04) | 1.03 (0.99, 1.07) |

1. Associations per coffee consumption increasing allele

2. Associations are per SD increase in genetic risk score

|  | **2 SNP genetic risk score** | | | **8 SNP genetic risk score** | | |
| --- | --- | --- | --- | --- | --- | --- |
|  | **N** | **Beta (95% CI)** | **P-value** | **N** | **Beta (95% CI)** | **P-value** |
| Low consumers (<=5 cups per day) | 57,144 | 0.06 (0.05, 0.07) | <0.001 | 55,716 | 0.07 (0.06, 0.08) | <0.001 |
| Medium consumers (>5-10 cups per day) | 49,353 | 0.06 (0.05, 0.07) | <0.001 | 48,129 | 0.07 (0.06, 0.08) | <0.001 |
| High consumers (>10 cups per day) | 5,365 | 0.02 (-0.09, 0.12) | 0.78 | 5,238 | 0.03 (-0.07, 0.14) | 0.52 |

**Table S4. Associations between coffee variants and combined coffee and tea consumption stratified by number of cups of coffee and tea consumed per day**

**Table S5. Mendelian randomisation analysis of the association of coffee genetic risk scores with water and total non-coffee or tea beverage consumption**

| **Method** | **Water (glasses)**  **(N = 110,820)** | **P-value** | **I-squared (%)** | **Total non-coffee or tea (portions)**  **(N =47,364)** | **P-value** | **I-squared (%)** |
| --- | --- | --- | --- | --- | --- | --- |
| Inverse variance weighted | -0.48 (-0.58, -0.38) | <0.001 | 68 (33,85) | -0.53 (-0.68, -0.38) | <0.001 | 50 (0,78) |
| MR Egger slope | -0.52 (-0.75, -0.28) | 0.002 | 72 (40,87) | -0.44 (-0.78, -0.09) | 0.02 | 55 (0,81) |
| MR Egger intercept | 0.003 (-0.016, 0.023) | 0.70 |  | -0.009 (-0.038, 0.020) | 0.48 |  |
| Median weighted | -0.49 (-0.61, -0.37) | <0.001 |  | -0.50 (-0.69, -0.33) | <0.001 |  |

Analyses performed using the 8 SNP genetic risk score. Coefficients are per additional cup of coffee consumed. MR Egger slope is the pleiotropy-adjusted estimate, MR Egger intercept is the test for directional pleiotropy. The I-squared statistic indicates the degree of variation between estimates from each SNP that is due to heterogeneity.

**Table S6. Mendelian randomisation analysis of the association of coffee genetic risk scores with daily alcohol consumption**

| **Method** | **Odds ratio for daily alcohol consumption (95% CI)**  **(N =111,407)** | **P-value** | **I-squared (%)** | **Log of alcohol consumption (units) (95% CI)**  **(N=85,410)** | **P-value** | **I-squared (%)** |
| --- | --- | --- | --- | --- | --- | --- |
| **Inverse variance weighted** | 1.17 (1.05, 1.32) | 0.01 | 82 (66,91) | 0.10 (0.04, 0.16) | 0.004 | 89 (80,94) |
| **MR Egger slope** | 0.93 (0.71, 1.22) | 0.55 | 82 (64,91) | -0.01 (-0.14, 0.12) | 0.83 | 89 (80,94) |
| **MR Egger intercept** | 1.02 (0.999, 1.05) | 0.06 |  | 0.01 (-0.0006, 0.12) | 0.06 |  |
| **Median weighted** | 1.11 (0.98, 1.25) | 0.10 |  | 0.07 (0.013, 0.131) | 0.02 |  |

Analyses performed using the 8 SNP genetic risk score. Coefficients are per additional cup of coffee consumed. MR Egger slope is the pleiotropy-adjusted estimate, MR Egger intercept is the test for directional pleiotropy. The I-squared statistic indicates the degree of variation between estimates from each SNP that is due to heterogeneity.

**Figure S1. Flowchart of study population**


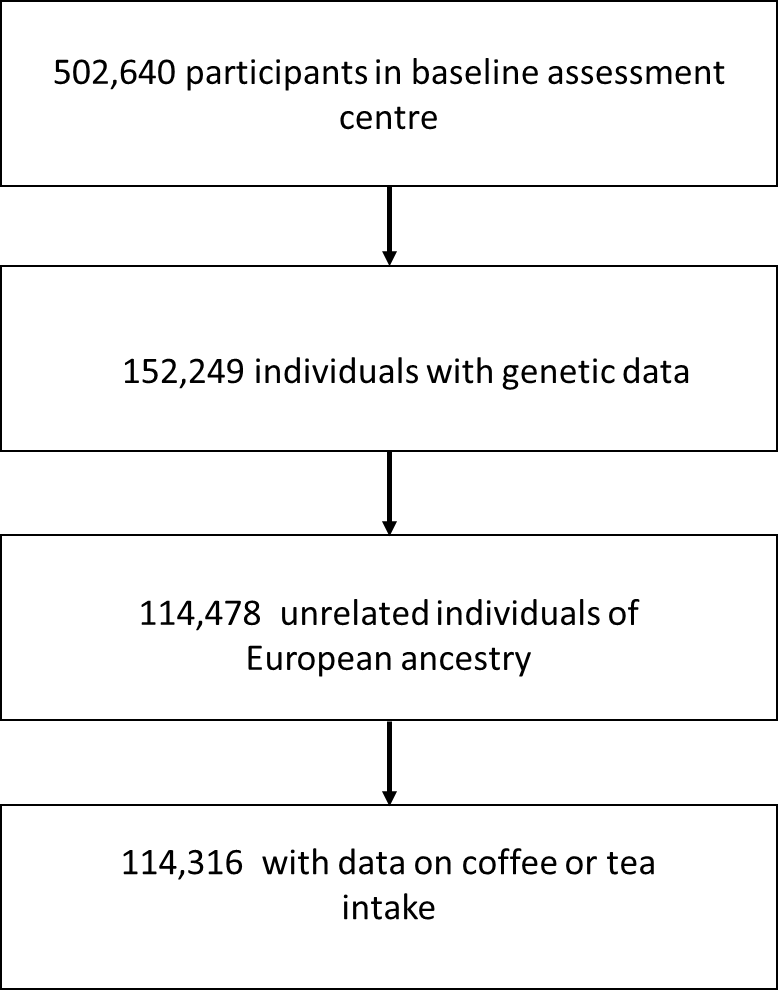


**Figure S2. Associations of individual SNPs with coffee consumption**

Adjusted for genetic principal components

**Figure S3. Association between 8 SNP weighted genetic risk score and types of drink in the full sample and dietary recall subset.**

Analyses restricted to consumers of each drink apart from the dietary recall total which included all respondents

**Figure S4. Association between 4 SNP weighted genetic risk score and types of drink in the full sample and dietary recall subset.**

Analyses restricted to consumers of each drink apart from the dietary recall total which included all respondents

**Figure S5.** **Associations between the 8 SNP genetic risk score and caffeinated and decaffeinated coffee.**

**Figure S6.** **Associations between the 4 SNP genetic risk score and caffeinated and decaffeinated coffee.**

**Figure S7. Association between genetic risk scores and continuous demographic and lifestyle factors stratified by combined coffee and tea consumption**

Low consumers (<=5 cups per day), Medium consumers (>5-10 cups per day), High consumers (>10 cups per day).

**Figure S8. Association between genetic risk scores and binary demographic and lifestyle factors stratified by combined coffee and tea consumption**

Low consumers (<=5 cups per day), Medium consumers (>5-10 cups per day), High consumers (>10 cups per day)

**Figure S9. Association between genetic risk scores and tea and coffee consumption stratified by smoking status.**

8 SNP score

2 SNP score

Adjusted for age, sex and genetic principal components
